# Molecular Epidemiology and Clone Transmission of Carbapenem-Resistant *Acinetobacter baumannii* in ICU Rooms

**DOI:** 10.3389/fcimb.2021.633817

**Published:** 2021-02-26

**Authors:** Xiufeng Zhang, Fangping Li, Furqan Awan, Hongye Jiang, Zhenling Zeng, Weibiao Lv

**Affiliations:** ^1^ South China Sea Institute of Oceanology, Chinese Academy of Sciences, Guangzhou, China; ^2^ Department of Biomedical Engineering, College of Engineering, The Hong Kong Polytechnic University, Hong Kong, China; ^3^ Guangdong Laboratory for Lingnan Modern Agriculture, Guangzhou, China; ^4^ College of Veterinary Medicine, Guangdong Provincial Key Laboratory of Veterinary Pharmaceutics Development and Safety Evaluation, National Risk Assessment Laboratory for Antimicrobial Resistance of Microorganisms in Animals, South China Agricultural University, Guangzhou, China; ^5^ Department of Clinical Laboratory, Shunde Hospital, Southern Medical University (The First People’s Hospital of Shunde), Foshan, China

**Keywords:** carbapenem-resistant, *Acinetobacter baumannii*, whole-genome sequencing, clone spread, nosocomial infection

## Abstract

Carbapenem-resistant *Acinetobacter baumannii* (CRAB) is a major cause of nosocomial infections and hospital outbreaks worldwide, remaining a critical clinical concern. Here we characterized and investigated the phylogenetic relationships of 105 CRAB isolates from an intensive care unit from one hospital in China collected over six years. All strains carried *bla_OXA-23_*, *bla_OXA-66_* genes for carbapenem resistance, also had high resistance gene, virulence factor, and insertion sequence burdens. Whole-genome sequencing revealed all strains belonged to ST2, the global clone CC2. The phylogenetic analysis based on the core genome showed all isolates were dominated by a single lineage of three clusters and eight different clones. Two clones were popular during the collection time. Using chi-square test to identify the epidemiologically meaningful groupings, we found the significant difference in community structure only existed in strains from separation time. The haplotype and median-joining network analysis revealed genetic differences appeared among clusters and changes occurred overtime in the dominating cluster. Our results highlighted substantial multidrug-resistant CRAB burden in the hospital ICU environment demonstrating potential clone outbreak in the hospital.

## Introduction

The spread of antibiotic-resistant bacteria poses a substantial public health crisis worldwide. *Acinetobacter baumannii*, known as a member of the ESKAPE group, is responsible for a vast array of nosocomial infections throughout the world (Santajit and Indrawattana, 2016). The extraordinary ability to survive in a harsh environment and to readily acquire antimicrobial resistance determinants has made *A. baumannii* as a public health threat (Garnacho-Montero and Timsit, 2019). Carbapenems are known as the frontline treatment for multidrug-resistant (MDR) *A. baumannii* infections. However, the widespread carbapenem resistant *A. baumannii* (CRAB) strains have caused a major concern worldwide due to the limited treatment choices (Doi, 2019). In 2017, the World Health Organization has listed CRAB as one of the most critical pathogens and the highest priority in new antibiotic development (Tacconelli et al., 2018).

Carbapenem resistance in *A. baumannii* is medicated by several coexisting mechanisms, while the most prevalent mechanism is associated with carbapenem-hydrolyzing enzymes (Codjoe and Donkor, 2018). There exist three different types of *ß*-lactamases leading to carbapenem resistance, such as ambler class A *β*-lactamases (*bla*_GES-14,_ *bla*_TEM_ *, bla*_SHV,_ *bla*_CTX-M_, and *bla*_KPC_), metallo-*ß*-lactamases (*bla*_IMP-like_, *bla*_VIM-like_, *bla*_SIM-1_, and *bla*_NDM-1_), and oxacillinases (*bla*_OXA-23-like_, *bla*_OXA-24-like_, *bla*_OXA-58-like,_ *bla*_OXA-143_, *bla*_OXA-235-like,_ *bla*_OXA-51-like_) (Evans and Amyes, 2014; Sawa et al., 2020). The major expression of OXA genes might be facilitated by insertion sequence elements (ISs), such as IS*Aba1*, IS*Aba4*, and IS*Aba125*, which provide an additionally strong promoter (Cardoso et al., 2016; Hamidian and Hall, 2018). In addition to the resistance, CRAB also carried a wide arsenal of virulence factors predisposing for a worsening course of disease (Morris et al., 2019; Ayoub Moubareck and Hammoudi Halat, 2020). Although virulence determinants in *A. baumannii* are incompletely understood, the genes related to biofilm formation, outer membrane proteins, surface glycoconjugates, micronutrient acquisition systems, and secretion systems were thought to be important for this pathogen to successfully infect its hosts (Harding et al., 2018). Hence, improved surveillance and understanding of CRAB is a key factor in reducing death tolls caused by *A. baumannii* infections.

Numerous nosocomial outbreaks caused by CRAB have been described in the world (Rodríguez et al., 2018; Teerawattanapong et al., 2018). In many cases, one or two epidemic strains were perceived in a certain epidemiological setting, particularly in intensive care units (ICUs) (Simo Tchuinte et al., 2019; Zhao et al., 2019). Molecular characterization reveals that clonal dissemination plays an important role in nosocomial CRAB outbreaks, and most of them belong to the global clones 1 and 2 (Douraghi et al., 2020). This might be due to the transfer of colonized patients who transmit these epidemic strains among hospitals. To date, the international clone ST2 was the most dominant type globally in all *A. baumannii* genomes sequenced (Levy-Blitchtein et al., 2018). Several studies showed the dissemination of CRAB isolates mainly harbored the *bla_OXA-23-like_* and belonged to ST2 from different countries (Karampatakis et al., 2017).

The recent expansion of *A. baumannii* sequenced genomes has permitted the development of large-array phylogenomic and phenotypic analyses, which can offer valuable insights into the evolution and adaptation of *A. baumannii* as a human pathogen (Yakkala et al., 2019; Makke et al., 2020). Though there are many studies on the molecular mechanisms of resistance and global epidemiology of CRAB, no study was designed to investigate the distribution, molecular epidemiology, phylogenetic relationships of CRAB recovered from ICUs. Therefore, we conducted a six-year-long longitudinal study at a tertiary care hospital in China, to study the epidemiology and characterization of CRAB on healthcare surfaces using whole-genome sequencing and bioinformatic analyses.

## Materials and Methods

### Strain Collection and Antimicrobial Susceptibility

All *A. baumannii* strains were part of the routine hospital laboratory procedure, and they were isolated from people who were hospitalized in ICU rooms between 2013 and 2018. Reference *A. baumannii* ATCC 19606 (strain M19606) was purchased from Guangdong culture collection center. For all isolates, the minimum inhibitory concentrations (MICs) of antibiotics were determined using a BD PhoenixTM 100 Automated Identification and Susceptibility Testing System (BD, USA) according to CLSI guideline (Clinical and Laboratory Standards Institute, 2018).

### Illumina Whole Genome Sequencing

Bacterial genomic DNA of *A. baumannii* strains were extracted using a HiPure Bacterial DNA Kit (MAGEN). The libraries were created using the VAHTS™ Universal DNA Library Prep kit for Illumina. Whole genome sequencing was carried out through an Illumina Hiseq 2500 system to obtain 2 × 150 bp reads. The sequence quality of the reads was evaluated using FastQC. Processed reads were *de novo* assembled into contigs with CLC Genomics Workbench 10.1 (CLC Bio, Aarhus, Denmark), and genomes were annotated by NCBI Prokaryotic Annotation Pipeline (PGAP).

### Taxonomic Assignment

FastANI v1.1 (https://github.com/ParBLiSS/FastANI) with the core algorithm of BLAST-based ANI solver was used to identify the species of all isolates. Reference *A. baumannii* ATCC 19606 participated in species identification as standard sample. Species were determined if the genome in question had >95% ANI index with the type genome (Richter and Rosselló-Móra, 2009). Finally, all the isolates sequenced in this study were used to construct a Triangular matrix, representing the product of the ANI and percent genome aligned (sp1).

### ARGs, VGs, IS Identification

Antibiotic resistance genes (ARGs) were annotated using the ResFinder BLAST identification program (https://cge.cbs.dtu.dk/services/ResFinder/) (Kleinheinz et al., 2014). Associated metadata was displayed as a color strip to represent bacterial isolate demographics and expected resistance to antibiotics. Virulence genes (VGs) were identified by the core database of VFDB (setA) (http://www.mgc.ac.cn/VFs/) using BLASTv2.7. ISs were annotated using IsFinder BLAST (https://www-is.biotoul.fr/index.php). The presence/absence matrix of ARGs, VGs, or ISs was visualized in pheatmap R-package (R).

### Clustering and Pan-Genome Analysis

Prokka v1.11 was used to produce gff file for the contig of *A. baumannii* genome. After that, Roary v3.8.0 and Mafft v7.0 were used to construct a core genome alignment (Katoh et al., 2002; Page et al., 2015). Core_genome_alignment.aln, the output file of Roary pipeline was conveyed to fastGEAR to identify instances of recombination within these samples. The recombinant regions were removed using the script of recombinant2out.py of Bacteriatool tool package (https://github.com/lipingfangs/Bacteriatool), which we designed for bacteria NGS data analysis including series of custom python scripts. Core genome alignment either removal of recombination regions or non-removal of recombination regions was used to generate a maximum likelihood tree with Iqtree v1.6.12 with 1,000 times bootstraps (Nguyen et al., 2015). The output newick file was visualized in iTOL (Letunic and Bork, 2016). *In silico* multilocus sequence typing (MLST) was performed with the MLST program. Lineages and clusters identified by hierBAPS during fastGEAR were also marked on the trees (Cheng et al., 2013).

### Clonality Analysis

Pairwise single nucleotide polymorphism (SNP) counts between all isolates in the recombinant corrected core genome alignment were calculated by snpdistance.py of Bacteriatool tool package. All paired distances <20 SNPs/a were considered as a clonotype for further study. Pairwise groupings were imported to R to compute the euclidean distance and visualize these groupings with R package ggplot2 (http://had.co.nz/ggplot2/).

### Spatiotemporal Clone Linkage and Population Dynamics Analysis

Based on SNP distance, R package vegan was used to PERMANOVA test in order to calculate the genetic diversity of the core genome between the clone types and the bayesian clusters in the sample community (Pimple, 2020). The basic R function chisq.test was adopted for examining the structural diversity of clonotype communities at different times and from different sources. The core genome alignment culling standard strain M19606 was conveyed to custom python script in order to extract the SNP sites which ignored site of miss. DnaSP was used for haplotype identification (Rozas et al., 2017). A median joining network of haplotypes was generated by the NETWORK program to indicate the relationship between each haplotype, clone type with time development (Bandelt et al., 1999). DnaSP and software Arlequin were used to dynamically analyze CRAB population by establishing mismatch curve (Das et al., 2002).

### Nucleotide Accession Number

These assemblies sequence data of *Acinetobacter baumannii* isolates were deposited in the GenBank database under BioProject accession: PRJNA541408, 541386, 541822, 542047, 542046, and 542048. In addition, the genome accession number for M19606, L9, and L40 was VAMZ00000000, VWQN00000000, and VWPZ00000000, respectively.

## Results

### ICU Rooms Had High Carbapenem-Resistant *A. baumannii* Burden

**Figure 1:**
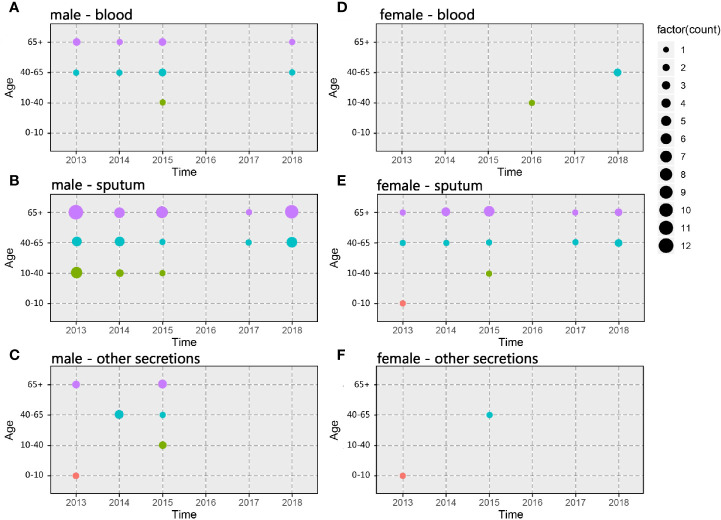
Spatiotemporal, demographic, and clinical characteristics of 105 isolates of Shunde hospital. **(A–C)** Sample distribution from male patients; **(D–F)** Sample distribution from female patients; the sampling time range was 2013–2018, and the patients were divided into four groups according to age: 0–10 years old, 10–40 years old, 40–65 years old and over 65 years old. The sampling source was divided into three groups: blood, sputum, and other secretions.

A total of 302 *A. baumannii* strains were isolated from the hospital ICU rooms, and 105 isolates showed resistant to imipenem and meropenem (**Supplementary Figure 1**). Then we choose all these CRAB strains and reference strain *A. baumannii* ATCC 19606 for phenotypic and genomic analysis. Hierarchical clustering of Hadamard values confirmed 106 isolates as *A. baumannii* (**Supplementary Excel S1**). The information about the source, year of isolation, and gender of patients of all CRAB isolates were shown in **Figure 1** and **Supplementary Excel S2**. Of these, 72.3 and 75.2% of 105 CRAB strains were from sputum and from patients with pulmonary infection, respectively. Most strains (53.3%) were isolated from older people, who are more than 65 years old.

### Single Lineages Dominated CRAB Population

**Figure 2:**
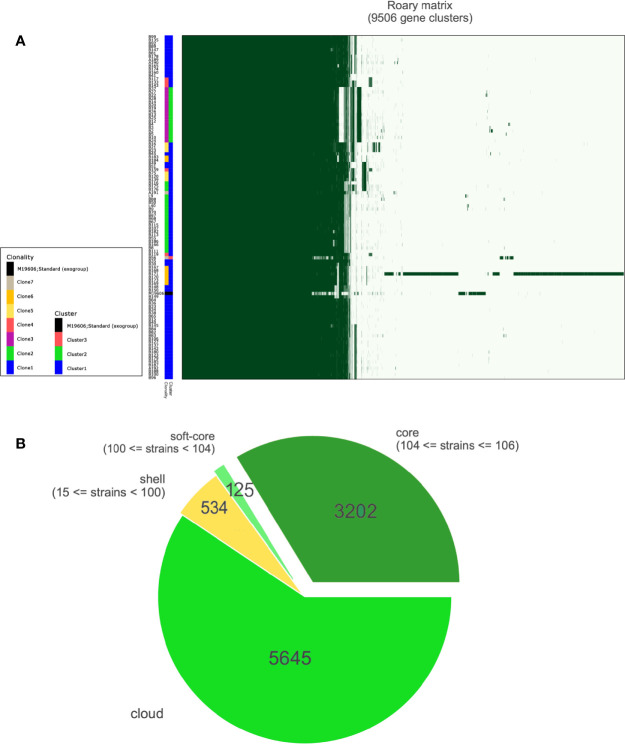
Results of pan-genomic analysis. **(A)** Matrix with the presence and absence of core and accessory genes. A total of 9,506 genes were clustered and annotated with different colors. different clonalities based on SNP distance and different clusters based on bayesian classification. **(B)** Pie chart shows different kinds of accessory genes; the number of core genes was 3,202.

Our results demonstrated that a single lineage represented of all CRAB strains collected over six years, which was composed of three clusters (**Figure 2A** and **Supplementary Figure 2**). Cluster 1, cluster 2, and cluster 3 contained 87, 17, and one isolates, respectively. Interestingly, all strains were assigned to ST2, which belonged to clonal complex 2 (CC2) and to international clone II. Besides, there were 9,506 genes in all these strains, and 99% of strains shared 3,202 core genes (**Figure 2B**). According to the core genome analysis, we found 1,746 recent homologous recombination sites in core genomes of all CRAB strains, and no ancestral homologous recombination sites were detected (**Supplementary Excel S3**).

### Three Clones Dominated CRAB Population

**Figure 3:**
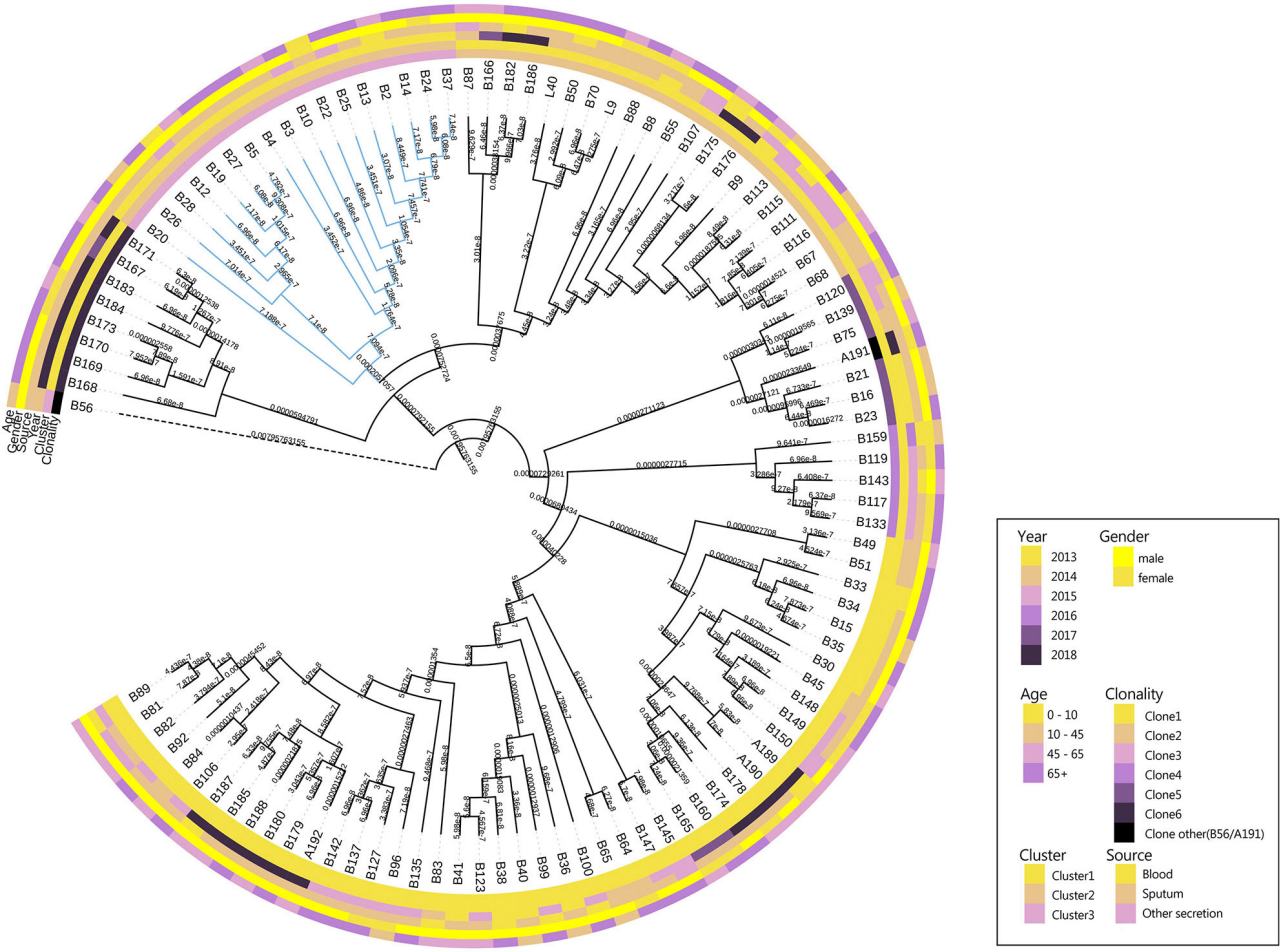
Maximum likelihood phylogenetic trees from core genome alignments of CRAB isolates. Phylogenetic tree constructed from the core genomic file excluding the standard strain M19606; colored annotations are added next to the main tree for sampling time and sources, patient ages and genders and cluster and clonality classification of 105 CRAB isolates.

**Figure 4:**
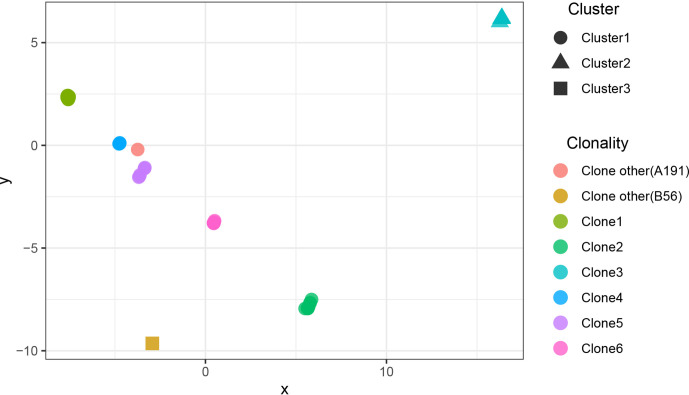
The scatter plots of SNP distance reduced dimension among CRAB isolates. The SNP distance matrix is standardized by R function scale and then the function dist (method = “euclidean”) is used to calculate the distance between variables. The results are visualized by using the function cmdscale and by Package ggplot2.

The results showed that CRAB had multimodal pairwise SNP distance distributions, indicating concordance between SNP distances and clustering on core genome phylogenetic trees (**Figures 3**, **4**). Eight clones (six clones and two clone others) were observed in CRAB strains. Of these, 43.8, 20, and 16.2% of all strains belonged to clone 1, clone 2, and clone 3, respectively. In addition, cluster 1 contained most clone groups, including clone 1, clone 2, clone 4, clone 5, clone 6, and clone other (A191), with strains collected from 2013 to 2018. Cluster 2 was only composed of clone 3, which with all the samples isolated from 2013. Cluster 3 only contained clone other (B56). The clone classification also conformed to genetic distance distribution matrix based on R package ape (**Supplementary Figure 3**). Permanova analysis of clone groups (including clone 1, 2, 3, 4, 5, 6) based on SNP distance showed that there were extremely significant differences among different clone groups (P < 0.01), which indicated that the classification is correct. However, permanova analysis between clone 1, 2, 3, 4, 5, 6 and other clone (A191 and B56) indicated that the significance difference among clones fluctuated (**Table 1**). Similarly, permanova analysis of cluster 1 and 2 demonstrated that there were extremely significant differences among clusters (p < 0.01). When cluster 3 was compared to cluster 1 or cluster 2, the results of permanova analysis were also different (p = 0.011 and p = 0.062, respectively) (**Table 2**).

**Table 1:** Results of permanova variance analysis among clonalities based on SNP distance (permutation = 10,000).

Group	SumsOfSqs	MeanSqs	F.Model	R^2^	Pr(>F)
Clone1/Clone2	13.8085505	13.8085505	270,758.7184	0.99976	0.001^***^
Clone1/Clone3	12.1801455	12.1801455	620,304.5903	0.9999017	0.001^***^
Clone1/Clone4	3.93263967	3.93263967	942.8360348	0.9505967	0.001^***^
Clone1/Clone5	4.71278078	4.71278078	2,737.589795	0.9820634	0.001^***^
Clone1/Clone6	6.47426112	6.47426112	17,532.50712	0.9970429	0.001^***^
Clone2/Clone3	9.15172599	9.15172599	156,083.8014	0.9997694	0.001^***^
Clone2/Clone4	3.8765566	3.8765566	7,205.820792	0.9966804	0.001^***^
Clone2/Clone5	4.33333067	4.33333067	8,269.488866	0.996986	0.001^***^
Clone2/Clone6	5.50382535	5.50382535	13,081.96483	0.9979403	0.001^***^
Clone3/Clone4	3.84579693	3.84579693	1,934,040.787	0.9999897	0.001^***^
Clone3/Clone5	4.34389168	4.34389168	673,012.9105	0.9999688	0.001^***^
Clone3/Clone6	5.40799762	5.40799762	2,447,588.684	0.9999906	0.001^***^
Clone4/Clone5	2.24636341	2.24636341	1,012.297445	0.9911877	0.002^**^
Clone4/Clone6	3.02713091	3.02713091	211,478.7872	0.999948	0.002^**^
Clone5/Clone6	3.17394014	3.17394014	10,754.74947	0.9988855	0.002^**^
Clone1/Clone other(B56)	0.54775975	0.54775975	12.16291191	0.2127763	0.018^*^
Clone1/Clone other(A191)	0.86621581	0.86621581	47.19376141	0.5118976	0.026^*^
Clone other(A191)/Clone2	0.90627404	0.90627404	131.2353356	0.8677558	0.044^*^
Clone2/Clone other(B56)	0.64017946	0.64017946	12.43001057	0.3832873	0.044^*^
Clone3/Clone other(B56)	0.93355985	0.93355985	4,476.969255	0.9964389	0.053
Clone other(A191)/Clone3	0.9409506	0.9409506	42,274.54415	0.9996217	0.06
Clone other(A191)/Clone6	0.87492897	0.87492897	2,740.37143	0.9974521	0.115
Clone6/Clone other(B56)	0.77836812	0.77836812	64.50872318	0.9021098	0.115
Clone5/Clone other(B56)	0.26723539	0.26723539	2.371854408	0.3217446	0.135
Clone other(A191)/Clone5	0.28517158	0.28517158	2.834401976	0.3617892	0.14
Clone other(A191)/Clone4	0.81305707	0.81305707	3,575.983694	0.9988827	0.166666667
Clone4/Clone other(B56)	0.74298762	0.74298762	212.3640798	0.9815126	0.166666667
Clone other(A191)/Clone other(B56)	0.5	0.5	NaN	1	NA

*means p < 0.05, where there is a statistical difference; **means p < 0.01, where there is a significant statistical difference; ***means p < 0.001, where there is a statistically significant difference.

**Table 2:** Results of permanova analysis of variance among clusters (permutation = 10,000).

Group	SumsOfSqs	MeanSqs	F.Model	R2	Pr(>F)
Cluster1/Cluster2	3.107777407	3.107777	47.80067	0.319095	0.001^***^
Cluster1/Cluster3	0.488015237	0.488015	4.655904	0.051358	0.011^*^
Cluster2/Cluster3	0.933559854	0.93356	4476.969	0.996439	0.062

*means p < 0.05, where there is a statistical difference; where there is a significant statistical difference; ***means p < .001, where there is a statistically significant difference.

### Temporal Distance Identified Relevant Epidemiology Groups

**Figure 5:**
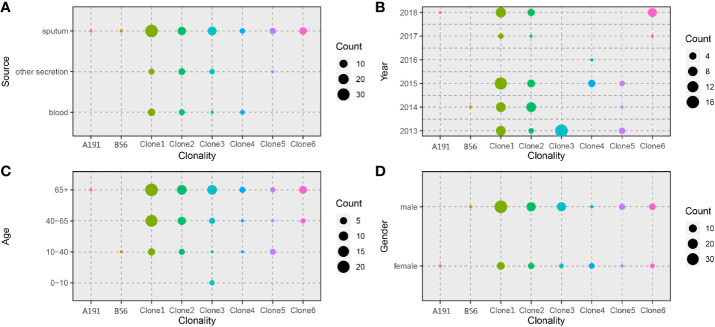
Distribution of CRAB clonalities in different spatial temporal, demographic, and clinical characteristics. **(A)** Distribution of CRAB clonalities in different sampling sources. **(B)** Distribution of CRAB clonalities in different sampling years. **(C)** Distribution of CRAB clonalities in different patient ages. **(D)** Distribution of CRAB clonalities in different patient genders.

We leveraged time, source, ages, and gender information to identify epidemiologically meaningful groupings with the chi-square test (**Figure 5**). The significant difference in community structure was only present in strains from separation time (p = 2.149e^-11^), absent in sampling sources (p = 0.2283), age of patients (p = 0.0931), and gender of patients (p = 0.0853).

**Figure 6:**
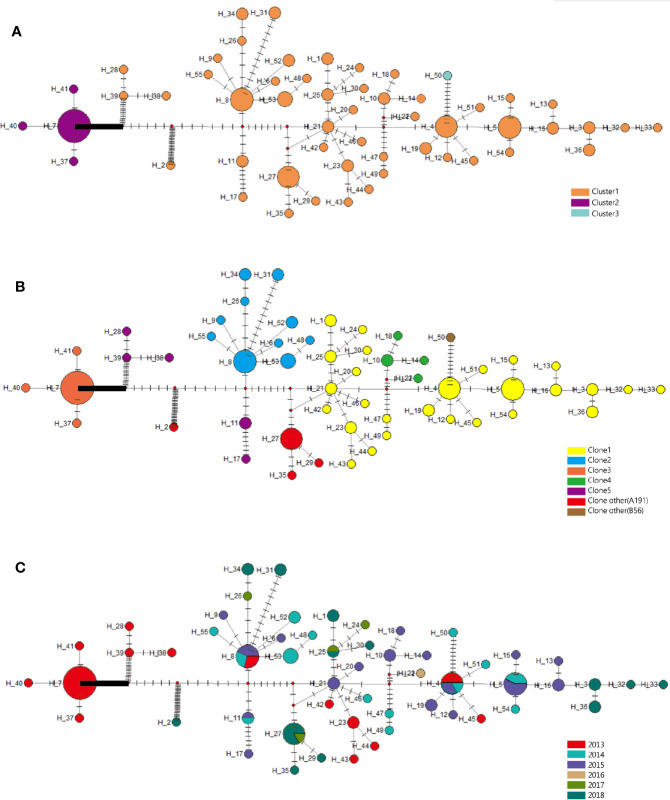
Evolutionary relationships and geographical distribution of 55 haplotypes of 105 isolates from Shunde hospital. **(A)** Circle colors represent different clusters based on bayesian classification. **(B)** Circle colors represent different clonalities based on SNP distance. **(C)** Circle colors represent sampling time. The density of those bars represents the genetic distance between the haplotypes; circle sizes represent the number of isolates in a haplotype.

Furthermore, clone 1 and clone 2 were all in high proportion of CRAB population across the whole collection time. In addition, there were 55 haplotypes found in CRAB samples, and the haplotype genetic relationship between different clone groups was quite different. There were large genetic differences among different bayesian clusters. Our results showed that cluster 2 (only exists in 2013) gradually disappeared over time, and cluster 1 subsequently became the dominant of the CRAB samples (**Figure 6**).

### Population Expansion in ICU CRAB Clusters

**Figure 7:**
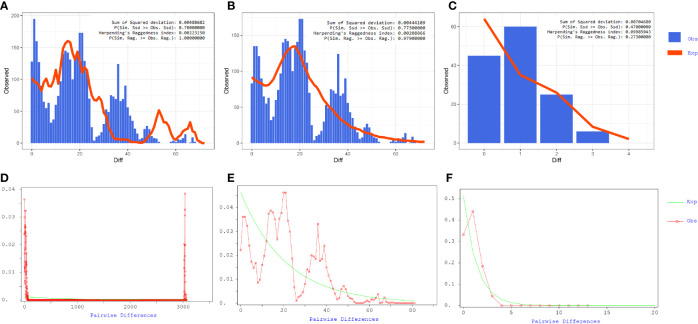
Mismatch distribution analyses of coding region alignment of 105 genomes from Shunde hospital. **(A–C)** Results of Mismatch distribution analyses by Alrequin. **(D–F)** Results of Mismatch distribution analyses by DnaSP.

Our results showed that the mismatch curve of total population (cluster 1 and cluster 2) and cluster 1 deviated from the expectation curve of assumed constant population size through DanSP analysis. The confidence of fitting curve of total population (p = 1) and cluster 1 (p = 0.97) (bootstrap = 1,000) indicated the population expansion was present (**Figure 7**).

### CRAB Isolates Shared High Genotypic and Phenotypic Resistance, Virulence Factors, and Insertion Sequences

**Figure 8:**
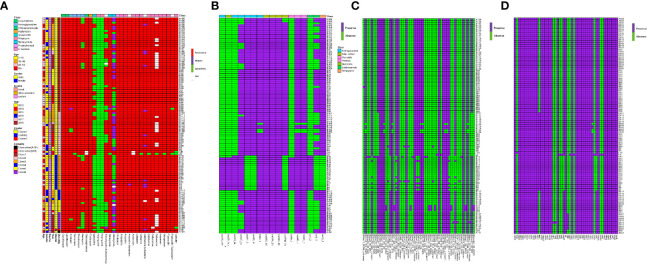
MIC test and ARGs, virulence genes (VGs), insertion sequence (IS) identification of 105 samples from ICU rooms. **(A)** Results of MIC test; class of antibiotics and resistance strength are annotated as colored bars next to the main heatmap. **(B)** Results of ARG identification based on Resfinder; **(C)** Results of IS based on ISfinder; **(D)** Results of VG identification based on VFDB.

Antibiotic susceptibility testing results revealed that all CRAB strains were resistant to at least three classes of antibiotics. All isolates showed 100% resistance against amoxicillin, ampicillin, cefoxitin, cefazolin, ceftazidime, gentamicin, chloramphenicol, and florfenicol (**Figure 8A**). The rate of other resistances was as follows: 99.05% for ampicillin/sulbactam, cefotaxime, cefepime, ciprofloxacin, tetracycline, fosfomycin, streptomycin, and piperacillin, 98.10% for aztreonam, levofloxacin and piperacillin/tazobactam, 97.14% for amikacin, 87.62% for ceforazone/sulbactam, 79.05 for neomycin, 69.52% for trimethoprim/sulfamethoxazole, 25.71% for tigecycline, and 12.38% for minocycline. None of them was resistant to polymyxins.

WGS analysis demonstrated that CRAB isolates harbored 17 unique ARGs. Carbapenemase genes *bla_OXA-23_*, *bla_OXA-66_* and cephalosporinase gene *bla_ADC-25_* were present in all strains. Genes encoding resistance to *β*-lactams (*bla_TEM-1D_*), aminoglycoside (*aac(6′)-Ib*, *aac(6′)Ib-cr*, *aph(3′)-Ia*, *aph(3′)-Ib*, *aph(6′)-Id*, *armA*, *aadA*), macrolide [*mph(E)*, *msr(E)*], amphenicol (*catB8*), tetracycline [*tet(B)*], and sulphonamide ARGs (*sul1, sul2*) were also detected (**Figure 8B**).

Given the extensive burden of high-risk ARGs found in CRAB isolates, we analyzed the existence of VFs and ISs. We identified 55 different types of ISs and 51 unique VFs (**Figures 8C, D** **)**. All the CRAB isolates had *bau (ABCDEF)*, *bas (basABCD*, *basFGHIJ)*, *csuABCD*, *pgaABCD*, *adeFGH*, *bfmRS*, *bap*, *entE*, *ompA*, and *plcD*. Interestingly, there were three isolates carrying unique VFs. The *pvdLDJI*, *phzA*, *pchE*, *plcH* genes were only present in L9 strain, the *hptB* and *clpC* genes in B171 strain, and the *fdeC* in L40 strain. ISs screening showed IS*Aba1/26/33*, IS*4*, IS*6*, IS*10*, IS*15*, IS*26*, IS*256* were found in all CRAB strains.

Besides, the hierarchical clustering of isolates based on ARGs, VFs, and ISs presence indicated that cluster was the major predictor of resistance, virulence, and IS patterns. Compared to clusters 1 and 3, ARGs like *aac(6′)-Ib*, *aac(6′)Ib-cr*, *aph(3′)-Ia*, *catB8*, *sul1* genes were nearly present in cluster 2 strains, while the genes *aadA*, *aph(6′)-Id*, *bla_TEM-1D_*, *tet(B)* were almost absent. In addition, the strains in cluster 2 were more likely to lack the *AbaIR*, IS*Aba46*-IS*66*, IS*Aba49*-IS*66*, IS*Aba16*-IS*66*, IS*Aba25*-IS*66*, IS*Alw34*-IS*66*, IS*Alw16*-IS*66* and IS*Vsa3*-IS*91*.

## Discussion

CRAB is a perilous nosocomial pathogen causing substantial morbidity and mortality, which is a severe health threat and economic burden (Carlos et al., 2017; Isler et al., 2018). Hospital setting is an important reservoir for CRAB transmission (Raro et al., 2017; Opazo-Capurro et al., 2019). This study shed some light on the population dynamics of CRAB and demonstrated the endemicity and dissemination of clonal complex 2/92 (Pasteur scheme/oxford scheme) in the ICU.

Hospitalized patients with open wounds and long hospital stays are among those who are vulnerable to *A. baumannii* infections, especially those hospitalized in intensive care units (ICU) (Sunenshine et al., 2007; Kamali et al., 2020). It was noted from this study that most patients were elder people who had weak natural defenses and long hospital stays. Besides, CRAB isolates are responsible for a wide range of infections, including pulmonary infection, abdominal infection, bloodstream infection, septic shock, and so on. Interestingly, almost all patients suffered from several diseases at the same time; the most common infection in this study was pulmonary infection, including pneumonia, respiratory failure. It was consistent with the fact that CRAB infection was often associated with pneumonia and bloodstream infection (Morris et al., 2019a; Moubareck and Halat, 2020; Sun et al., 2020).

Carbapenem resistance in *A. baumannii* is mainly based on the production of carbapenemases (Hsu et al., 2017). In our study, all strains harbored the *bla_OXA-23_* and *bla_OXA66_* responsible for carbapenem resistance. This parallels previous investigations that *bla_OXA-23_* was the most widely reported gene, and *bla_OXA66_* was the most common variant of OXA genes (also known as *bla_OXA51-like_*) (Rao et al., 2020). The increased antibiotic resistance in *A. baumannii* is largely owing to the actions of mobile genetic elements, activation of intrinsic resistance mechanisms such as the chromosomal *β*-lactamases, *bla_ADC_*, and the presence of efflux pumps (Kumburu et al., 2019). This study showed that a large diversity of ISs and AbaR-type genomic resistance islands (AbaRI) was present. It was noteworthy that all strains carried IS*Aba1/26/33*, IS*4*, IS*6*, IS*10*, IS*15*, IS*26* and IS*256*. Insertion of IS*Aba1* in the *bla_OXA-23_* promoter sequence has been reported to be associated with overexpression of *bla_OXA-23_*, *bla_OXA-51_* and carbapenem resistance phenotypes in *A. baumannii* (Lee et al., 2017). In addition, AdeFGH RND efflux pumps were also identified in all CRAB strains, which have been reported to play a major role in acquired resistance and may also be associated with carbapenem non-susceptibility (Coyne et al., 2010; Yoon et al., 2015). In accordance with others reports, these genetic structures could disseminate antibiotic resistance determinants between pathogens and thus compromise antibiotic treatment, including those prescribed antibiotics (Partridge et al., 2018; Abdi et al., 2020). In this study, the CRAB isolates had a high ARG burden and were phenotypically resistant to multiple classes of antibiotics commonly used in the clinic. Most notable is the present of 28 CRAB isolates showing resistance to tigecycline, which limited treatment options for their infections.

It is largely believed that drug resistance and virulence factors have enabled *A. baumannii* to thrive in unfavorable conditions, particularly in nosocomial environment (Morris et al., 2019b). Interestingly, the virulence genes are conserved in all CRAB strains. The presence of virulence factors, such as *bap*, *csuABCD*, *pgaABCD*, *bfmRS*, *entE*, *ompA*, and *plcD* suggests the infectious property of these strains (Choi et al., 2010; Liu et al., 2018). The Csu pili (*csuA/BABCDE*, regulated by the BfmRS two component regulatory system), biofilm-associated proteins (*bap*), the production of polysaccharide poly-N-acetylglucosamine (*pgaABCD*) were proved to be implicated in the biofilm formation, maintenance, and maturation (Eze et al., 2018; Kim et al., 2019). Studies have found that biofilm formation is more strongly associated with MDR *A. baumannii* strains or clinical isolates. Besides, most of the acinetobactin biosynthetic genes (*basABCDFGHIJ*) or the genes involved in acinetobactin uptake (*bauABCDEF*), siderophore efflux system (*barAB*) were all present in CRAB isolates (Hasan et al., 2015). The presence of the most important virulence genes in all CRAB isolates is in agreement with their clinical importance and confirmed the complexity of clinical infections caused by these strains.

There have been many reports of clonal outbreaks of *A. baumannii* mainly related to three international clone lineages (European clones I, II, and III) (Zarrilli et al., 2013). For the MLST scheme, most outbreak strains belong to CC1 and CC2, corresponding to European clones I and II, respectively. So far, ST2 type as the most common ST in CC2 has been reported in many countries, including China. The results of this study showed that all CRAB isolates belonged to ST2, suggesting at least an outbreak situation in ICU rooms. Of note, *bla_OXA-23_* and *bla_OXA66_* positive ST2 isolates were responsible for many outbreak infections (Huang et al., 2019). Besides, ST2, ST25, and ST78 strains produced more biofilm than other STs, which was consistent with our strains carrying several virulence factors responsible for biofilm formation (Raible et al., 2017). This fact has potentially led to more successful colonization of the clinical environment over time.

Through core genome phylogenetic analysis, we found that our CRAB isolates are dominated by single lineage. Previous report of *A. baumannii* and *E. faecium* isolates from Pakistan and the USA hospital system showed they were similarly dominated by single lineages (D'Souza et al., 2019). Contrastingly, these strains were composed of three clusters and eight clones. A previous study reported that six of the eight Danish *A. baumannii* isolates were located on three distinct clusters based on core genome phylogenetic analysis (Hammerum et al., 2015). Two clusters have the strains assigned to ST195 and ST208, which both belonged to ST2. In our study, cluster 1 and cluster 2 were the main clusters, clone 1 and clone 2 were found in 5/6 time points during our six-year collections. This observation, particularly in the light of the time separation between isolation events, suggests existence of specific CRAB strains in the Shunde hospital and some predominant clones circulating in this North China region.

It was noted that changes in clone groups were happened over the years. Clones 3, 4, and 5 disappeared in 2014, 2017, and 2016, respectively, whereas clone 6 emerged in 2017. This might also be caused by the insufficient isolates collected from 2016 to 2017. Hence, further study to explain this is needed. In addition, changes in main clone groups were found in 2018 due to the hospital’s move to another place. However clone 1 and clone 2 were still found in 2018, which indicated that the clone outbreak may be associated with doctors and nurses or with the medical equipment. Especially, there were several clones coexisting in the surgical observation area of open environment, where patients stayed for one or two days after the operation (**Supplementary Figure 4**). This increased the risk of cross infection among patients, as well as the main cause of nosocomial infection and bacteria outbreak. This phenomenon was improved evidently when increasing the number of inpatient wards and the closure of surgical observation area. These results aroused us to consider the effect of the hospital environment on nosocomial bacterial infections. Previous studies have reported that *A. baumannii* were isolated from medical apparatus, water systems, and handwashing sinks in the hospital environment, including ICUs and surgical wards (Umezawa et al., 2015).

According to the haplotype and the median-joining network analysis, we found large genetic differences among different clusters. Of note, cluster 2 was the dominant among all CRAB isolates at first (in 2013) and then substituted by cluster 1 between 2014 and 2018. These two clusters also presented different degrees of population expansion. There was no study that focused on population variation analysis of the same ST CRAB strains. Isolates in cluster 1 and cluster 2 were found to possess obvious different resistance, virulence, and IS patterns, which further confirmed the genetic differences between the two clusters. Compared to cluster 1, cluster 2 contained relatively little IS and resistance gene types. This might led to the subsequent prevalence of cluster 1 in Shunde hospital. The origin of these clusters is unknown, but it seems likely that they dwell in the hospital environment and cause infections over an extended period of time. These isolates tended to be MDR and hence could persist for a long time if the reservoir is not destroyed, serving as a potential source for nosocomial infection and recurrent outbreaks. In conclusion, based on the molecular epidemiology and genomics data, we confirm that polyclonal nature of the CRAB outbreak in hospital mainly caused by ST2 clone carrying *bla_OXA-23_* and *bla_OXA-66_*.

## Conclusion

We found the wide dissemination and clone outbreak of CRAB ST2 in ICU rooms of hospital. All strains had high genotypic and phenotypic resistance burden and were dominated by a single lineage of three clusters and eight different clones. The main cluster changed over years, and CRAB strains with different genetic backgrounds presented different degree of population expansion.

## Data Availability Statement

The datasets presented in this study can be found in online repositories. The names of the repository/repositories and accession number(s) can be found in the article/supplementary material.

## Ethics Statement

This project including the samples used in this study were approved by the medical ethics committee of Foshan Shunde First People’s Hospital. All patients have been informed of the purpose of collecting samples and our research has obtained the consent from all patients. Written informed consent to participate in this study was provided by the participants’ legal guardian/next of kin.

## Author Contributions

WL and ZZ conceived this study and designed the experiments. XZ and FL drafted the manuscript. WL offered all the CRAB strains. XZ, FL, and FA performed the bioinformatic analysis. HJ performed antimicrobial susceptibility tests for all CRAB strains. All authors contributed to the article and approved the submitted version.

## Funding

This work was supported by the National Natural Science Foundation of China (Grant No. 31672608).

## Conflict of Interest

The authors declare that the research was conducted in the absence of any commercial or financial relationships that could be construed as a potential conflict of interest.
