# Supplementary material for: Molecular Epidemiology and Clone Transmission of Carbapenem-Resistant Acinetobacter baumannii in ICU Rooms
Source: Front Cell Infect Microbiol. 2021 Feb 26;11:633817. doi: 10.3389/fcimb.2021.633817 (PMC7952536; doi:10.3389/fcimb.2021.633817)
Supplement: Supplementary file 1 [file DataSheet_1.docx]

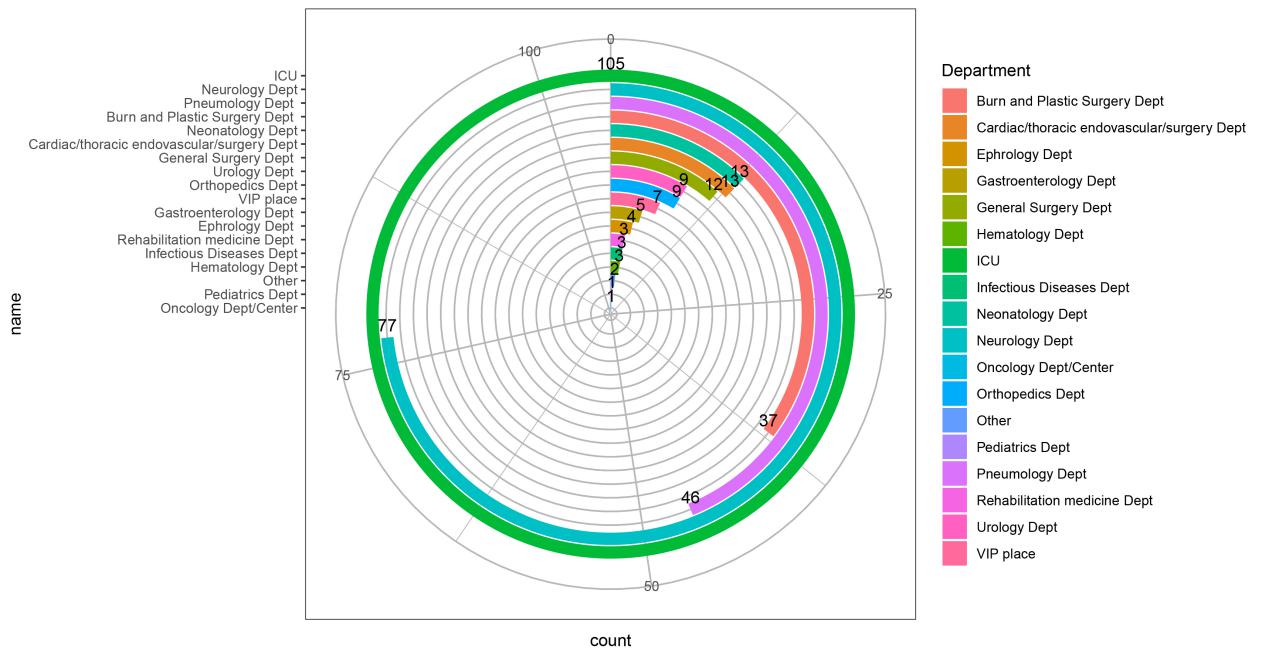


**Fig S1** Distribution of CRAB isolates in different departments.


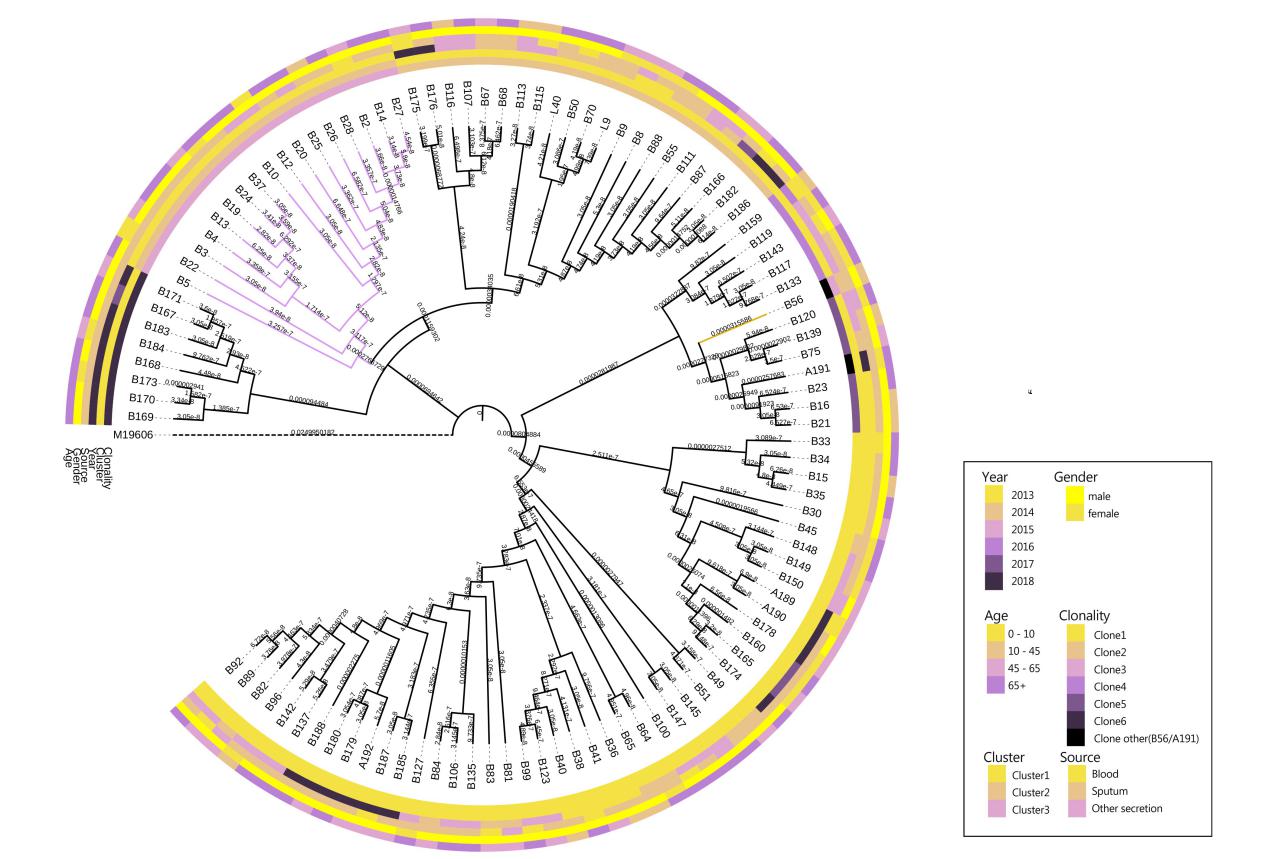


**Fig S2** Maximum likelihood phylogenetic trees from core genome alignments of CRAB isolates

Phylogenetic tree constructed from the core genomic file including the standard strain M19606 as out group


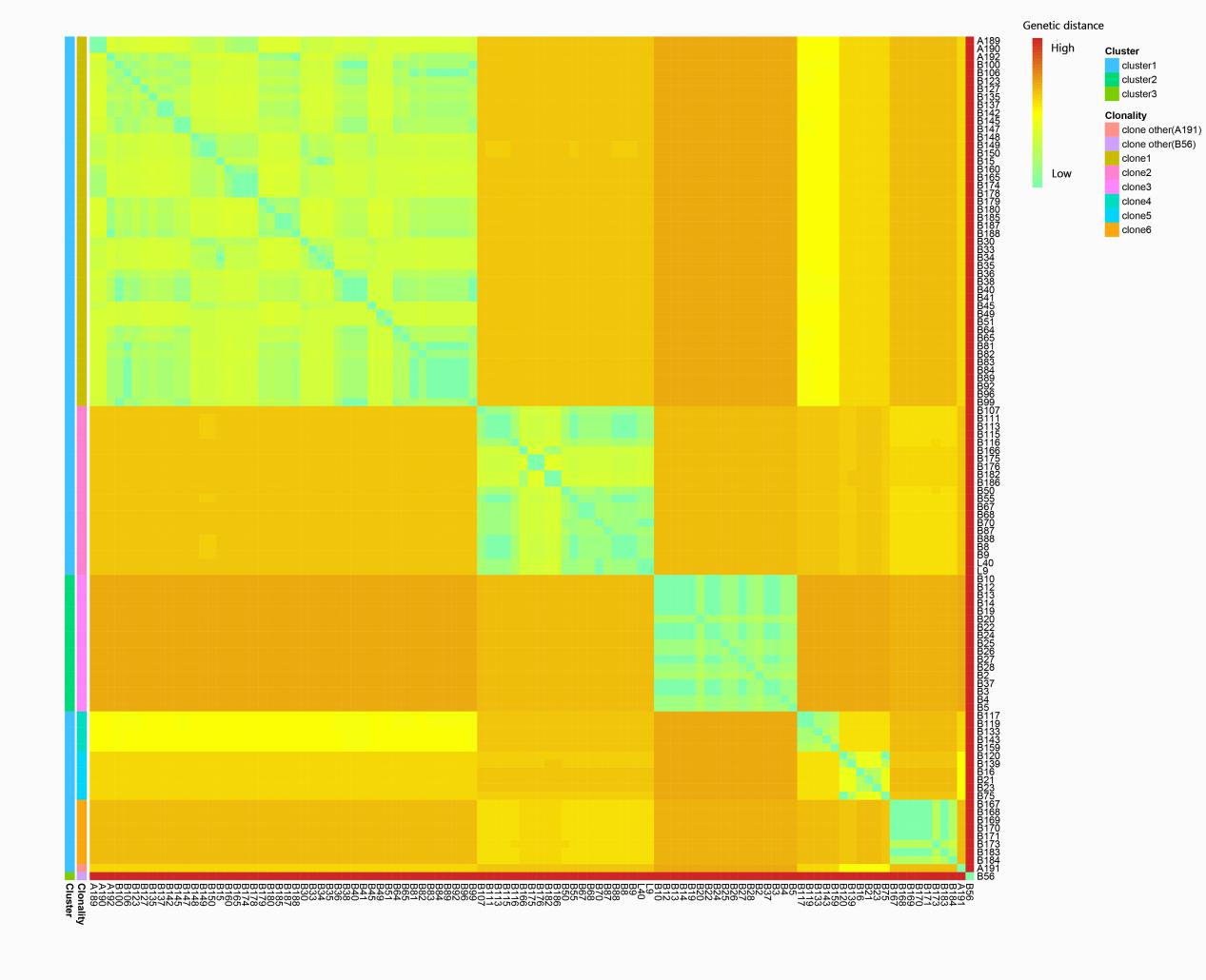


**Fig S3** The result of genetic distance distribution matrix based on R package Ape.


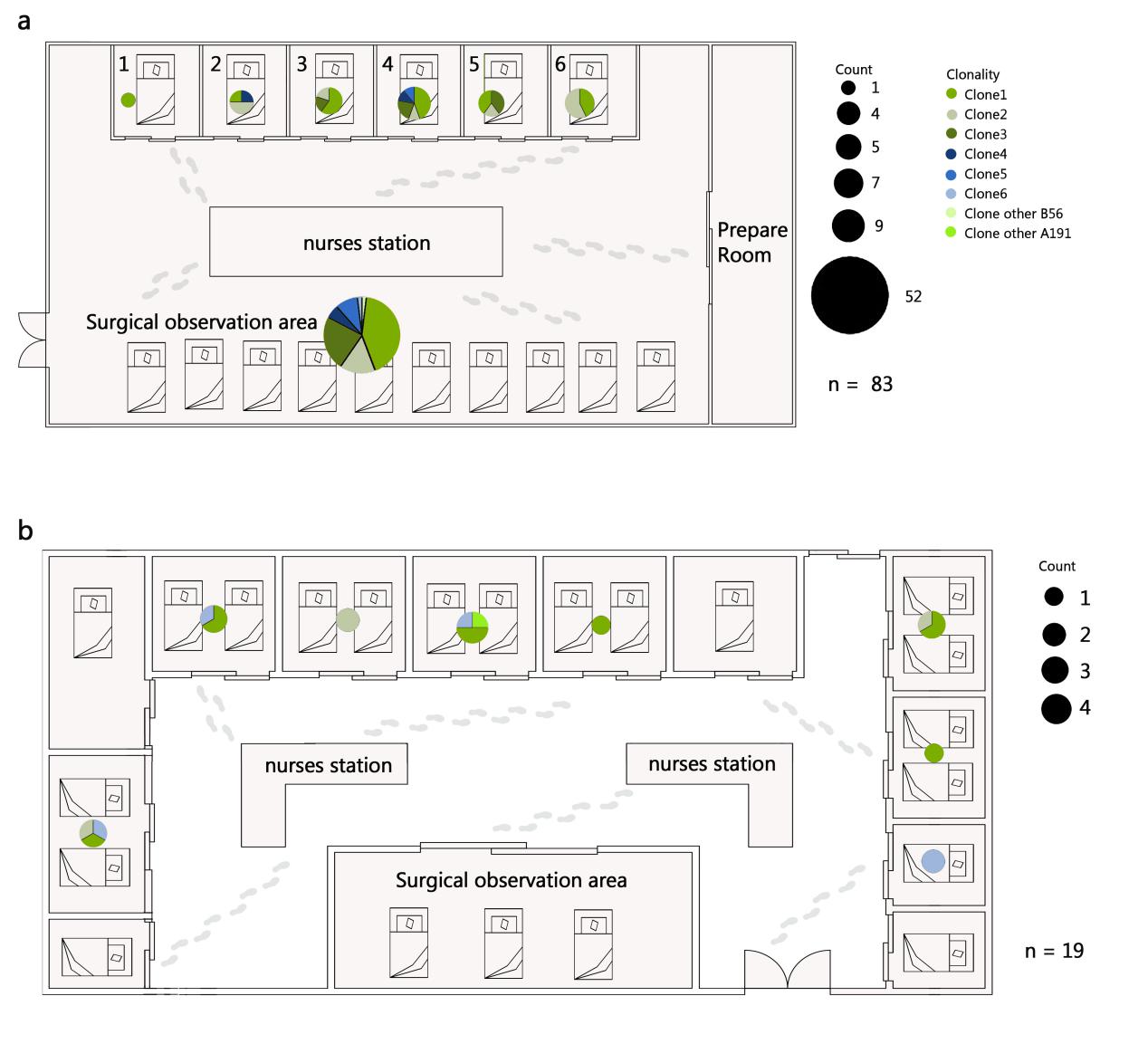


**Fig S4** Distribution of CRAB clonalities in different ICU rooms

**a** Distribution of CRAB clonalities in different ICU rooms from 2013 to 2017. **b** Distribution of CRAB clonalities in different ICU rooms in 2018.
